# Octacalcium phosphate collagen composite stimulates the expression and activity of osteogenic factors to promote bone regeneration

**DOI:** 10.1002/term.2969

**Published:** 2019-11-12

**Authors:** Atsumu Kouketsu, Keiko Matsui, Tadashi Kawai, Yushi Ezoe, Toshiki Yanagisawa, Ayato Yasuda, Tetsu Takahashi, Shinji Kamakura

**Affiliations:** ^1^ Division of Oral and Maxillofacial Surgery, Department of Oral Medicine and Surgery Tohoku University Graduate School of Dentistry Sendai Japan; ^2^ Division of Oral and Maxillofacial Surgery, Department of Medicine of Sensory and Motor Organs, Faculty of Medicine University of Miyazaki Kiyotake Japan; ^3^ Division of Oral and Maxillofacial Surgery, Department of Oral and Maxillofacial Reconstructive Surgery, School of Dentistry Iwate Medical University Morioka Japan; ^4^ Bone Regenerative Engineering Laboratory, Graduate School of Biomedical Engineering Tohoku University Aoba‐Ku Japan

**Keywords:** bone regeneration, bone tissue engineering, octacalcium phosphate collagen composite, osteogenic factors

## Abstract

**Objective:**

This study investigated the bone regenerative properties of an octacalcium phosphate collagen composite (OCP/Col) in a rat calvarial bone defect model.

**Design:**

An OCP/Col or *β*‐tricalcium phosphate (*β*‐TCP)/Col disk was implanted into the critical‐sized calvarial defects and fixed 2 or 4 weeks later. The radiopacity of defects was examined after disk implantation by the radiographic examination and micro‐computed tomography (*μ*‐CT). Immunohistochemical and histochemical analyses were carried out to assess the bone matrix maturation, neovascularization, and osteoclast and osteoblast distribution in the neonatal bone.

**Results:**

Radiographic and *μ*‐CT examination of the area of implanted OCP/Col indicated the newly formed bone and no difference from those of the original bone. Osteopontin, osteocalcin, Runt‐related transcription factor 2, type 1 collagen, vascular endothelial growth factor, and alkaline phosphatase or tartrate‐resistant acid phosphatase in the newly formed calvarial bone and the surrounding connective tissue were detected by immunohistochemistry and histochemistry. Biomarker expression was not significantly elevated at the defect site; the area of which was calculated by dividing the distance from the healthy bone margin or calvarium and dura mater surface. There was no difference in the expression of these biomarkers in the OCP/Col group at 2 and 4 weeks after surgery. In addition, the expression levels of all markers were higher in the OCP/Col group than in the *β*‐TCP/Col group at 2 and 4 weeks after surgery.

**Conclusions:**

The OCP/Col as a bone regeneration material not only exhibits osteoconductive activity that is dependent on residual healthy bone tissue, but also has osteoinductive capacity, which promotes angiogenesis and osteogenic cell invasion from host tissue into the bone defect.

## 1 INTRODUCTION

Technologies such as bone grafts and synthetic materials that are typically used to repair bone defects can be used in conjunction with standard surgical methods. Numerous materials have been developed to regenerate bone tissue in order to provide an alternative to autologous bone grafting, which has certain disadvantages such as limited tissue availability and morbidity associated with harvesting the bone from a second operative site (LeGeros, 2002). Calcium phosphates, such as hydroxyapatite (HA: Ca_10_(PO_4_)_6_(OH)_2_) and *β*‐tricalcium phosphate (*β*‐TCP: Ca_3_(PO_4_)_2_), are standard materials that are typically used for bone regeneration. Their synthetic forms are widely employed as bone substitutes in the clinical settings (Habraken, Habibovic, Epple, & Bohner, 2016; Kokubo, Kim, & Kawashita, 2013).

Octacalcium phosphate (OCP: Ca_8_H_2_(PO_4_)_6_·5H_2_O) is a precursor of biological apatite present in bones and teeth (Brown, Smith, Lehr, & Frazier, 1962). It has been detected in porcine enamel (Tohda, Yamada, Yamaguchi, & Yanagisawa, 1997), human dentin (Bodier‐Houlle, Steuer, Voegel, & Cuisinier, 1998), and mouse calvarial bone (Crane, Popescu, Morris, Steenhuis, & Ignelzi, 2006) as an intermediate in the formation of apatite matrices. The bone formation capacity of this synthetic bioresorbable material is superior to that of other bone substitutes (Kamakura et al., 2002; Kamakura, Nakajo, Suzuki, & Sasano, 2004; Kamakura et al., 1997). An OCP and collagen composite (OCP/Col) has been developed to overcome the moldability and the handling limitations associated with OCP (Kamakura, Sasaki, Honda, Anada, & Suzuki, 2006). This composite regenerates the bones more effectively than the OCP granules and other commercialized bone substitutes, achieving stable bone regeneration without cell transplantation (Iibuchi et al., 2010; Kamakura et al., 2006; Kamakura et al., 2007; Matsui et al., 2010; Miura et al., 2012; Tanuma et al., 2012). OCP/Col is expected to enhance the physiological remodeling of the bone and is easy to handle as well as cost effective.

The safety and efficacy of OCP/Col for bone regeneration in tooth extraction sockets and cyst holes have been demonstrated in clinical studies (Kawai et al., 2014; Kawai et al., 2016; Kawai et al., 2017). A sponsor‐initiated clinical trial of OCP/Col that was registered with the International Clinical Trials Registry of the World Health Organization (JPRN‐UMIN000018192) was recently completed, and commercialization of OCP/Col for the treatment of bone defects in the oral region including sinus floor elevation and alveolar clefts is expected in 2019.

Although considerable progress has been made towards employing OCP/Col in a clinical setting, the underlying mechanisms and the factors involved in the bone regeneration process induced by OCP/Col are yet to be fully elucidated; this is important for establishing the mass limitation in bone regeneration. Our previous study suggested that angiogenesis occurred earlier than the new bone formation induced by OCP/gelatin composite (Kurobane et al., 2019), but these results were insufficient to demonstrate the relationships among other bone formation markers and angiogenesis factors in OCP/Col treatment. Therefore, the present study investigated whether OCP/Col has positive effects on bone regeneration by evaluating the expression and activity of cells or molecules related to bone formation during the critical early phase of healing, by immunohistochemistry and enzyme histochemistry, in a rat calvarial bone defect model.

## 2 MATERIALS AND METHODS

### 2.1 Animals

Twelve‐week‐old male Wistar rats (SLC Corp., Hamamatsu, Japan) weighing 250–300 g were used for experiments. The principles of laboratory animal care as well as national laws were followed. All procedures were approved by the Animal Research Committee of Tohoku University.

### 2.2 Preparation of OCP/Col and *β*‐TCP/Col

We previously described the preparation of OCP/Col (Kamakura et al., 2006); OCP was prepared by direct precipitation (Suzuki, Nakamura, Miyasaka, Kagayama, & Sakurai, 1991), and sieved granules (particle size: 300–500 μm) were sterilized by heating at 120°C for 2 hr. Sintered *β*‐TCP was purchased from a commercial source (OSferion; Olympus, Tokyo, Japan), and sieved granules (particle size: 300–500 μm) were prepared for the fabrication of *β*‐TCP/Col. Collagen was prepared from Nippon Meat Packers collagen porcine skin (NMP collagen PS; Nippon Meat Packers, Tsukuba, Japan), which is a lyophilized powder of pepsin‐digested atelocollagen isolated from the porcine dermis. The NMP collagen PS was dissolved in distilled water and was adjusted to a final concentration of 3% at pH 7.4. OCP and *β*‐TCP granules were mixed with concentrated collagen. The OCP/Col and *β*‐TCP/Col mixtures were lyophilized and molded into the disks (diameter: 9 mm, thickness: 1 mm). Each prepared disk contained the same volume of OCP (10.5 mg) or *β*‐TCP (32.5 mg). Disks were subjected to dehydrothermal treatment (150°C, 24 hr) in a vacuum drying oven (DP32; Yamato Scientific, Tokyo, Japan) and subsequently sterilized by electron beam irradiation (15 kGy).

### 2.3 Implantation procedure

The procedure for the implantation of OCP/Col and β‐TCP/Col disks has been previously described (Iwai et al., 2018; Kamakura et al., 2007). The experimental animals were anesthetized intraperitoneally with 0.15 mg/kg dexmedetomidine hydrochloride (Domitor) (Nippon Zenyaku Kogyo Co., Ltd., Koriyama, Fukushima, Japan), 2.5 mg/kg butorphanol tartrate (Vetorphale) (Meiji Seika Pharma Co., Tokyo, Japan), and 2 mg/kg midazolam (Dormicum) (Astellas Pharma, Tokyo, Japan). The periosteum of the calvarium was ablated; a full‐thickness standard trephine defect, 9 mm in diameter, was made in the calvarium without damaging the dura mater; and an OCP/Col or β‐TCP/Col disk was then implanted and placed centrally into the defect.

**Figure 1:**
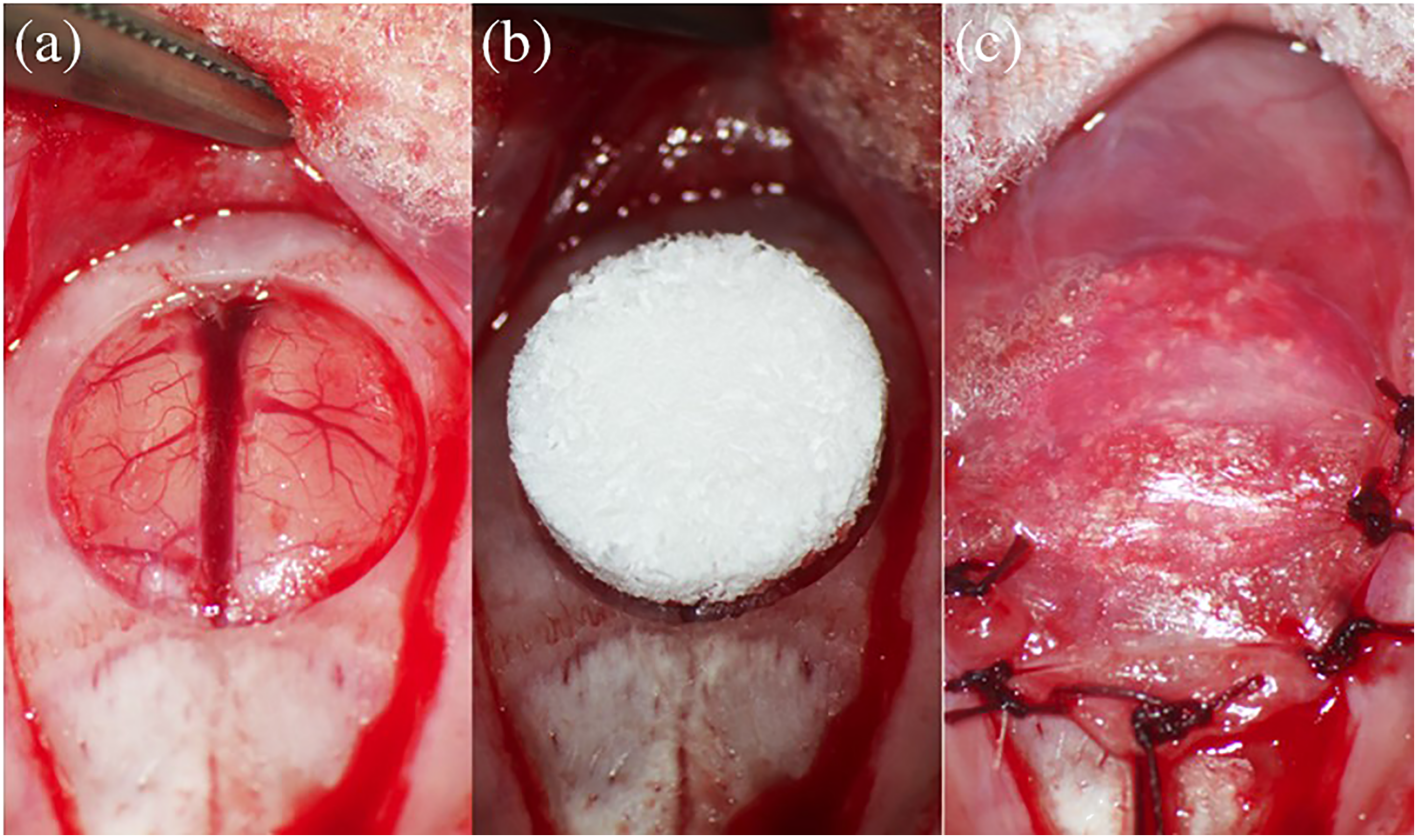
Implantation procedure. a, After ablating the periosteum of the calvarium, a full‐thickness standard trephine defect with a diameter of 9 mm was created. a, After saline irrigation of the defect, an octacalcium phosphate collagen composite (OCP/Col) or *β*‐tricalcium phosphate collagen composite (β‐TCP/Col) disk was implanted into the defect. c, The ablated periosteum was repositioned and sutured [Colour figure can be viewed at http://wileyonlinelibrary.com]

The defects were filled with implants of the same volume. Five rats per group and time point were implanted and treated with OCP/Col or β‐TCP/Col and the ablated periosteum and skin were repositioned and sutured (Figure 1). After surgery, the rats were subcutaneously injected with 15 mg/kg cephalexin to prevent infection. The experimental animals were euthanized by an intraperitoneal injection of an overdose of sodium pentobarbital after 2 or 4 weeks of surgery.

### 2.4 Micro‐computed tomography (*μ*‐CT) analysis

The radiopacity of defects was examined in live rats 2 and 4 weeks after disk implantation by *μ*‐CT (Latheta LCT‐200; Hitachi Aloca Medical, Tokyo, Japan). Images were acquired at a low voltage and at a slice thickness of 120 μm. To prevent excessive movement during scanning, rats were anesthetized by an intraperitoneal injection of sodium pentobarbital (50 mg/kg) supplemented with ether inhalation.

### 2.5 Radiographic analysis

After sacrifice, the samples were resected with the surrounding bone, and the tissue was fixed with 4% paraformaldehyde in 0.1 M phosphate‐buffered saline (pH 7.4). Next, the radiography of the skulls was performed with a microradiography unit (Softex CMR Unit; Softex, Tokyo, Japan) under standard conditions (20 kV, 5 mA, 1 min).

### 2.6 Tissue preparation and histopathological analysis

After radiography, samples were decalcified in 10% EDTA in 0.01 M phosphate buffer (pH 7.4) at 4°C. Tissue samples were fixed in 10% buffered formalin for several days and the center of the defect was removed and embedded in paraffin; 3 μm‐thick coronal sections were used for hematoxylin and eosin (HE) and elastica‐Masson (EM) staining and immunohistochemical analysis.

### 2.7 Immunohistochemistry and enzyme histochemistry by alkaline phosphatase/tartrate‐resistant acid phosphatase (ALP/TRAP)

**Figure 2:**
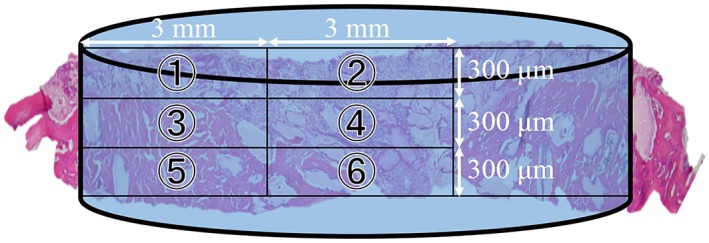
Compartments for the microscopic analysis of regenerated bone in critical‐size defects. Specimens were divided into the implant left outer and medial six sections and evaluated at the lateral plane (1–6): 0–300, 300–600, and 600–900 μm from the calvarial surface and 0–3 and 3–6 mm from the bone defect edge [Colour figure can be viewed at http://wileyonlinelibrary.com]

To visualize the new bone formation and the related cells or molecules by ALP/TRAP staining and immunohistochemistry, the observation area was divided into six parts; and the lateral plane slices were evaluated as follows (Figure 2): sections were obtained at 0–300, 300–600, and 600–900 μm from the calvarial surface and each of these layers was divided into the implant left outer and medial parts (0–3 and 3–6 mm, respectively, from the trephine defect margin).

Bone regeneration was detected by immunohistochemistry using antibodies against osteopontin (OPN) (ab63856), osteocalcin (OCN) (ab13420), Runt‐related transcription factor 2 (RUNX2) (ab23981), and collagen I (ab34710) (all from Abcam, Cambridge, UK) and vascular endothelial growth factor (VEGF) (Santa Cruz Biotechnology, Santa Cruz, CA, USA; C‐1, sc‐7269). The ALP/TRAP activity was detected with the ALP/TRAP Staining Kit (Fujifilm Wako Pure Chemical Industries, Osaka, Japan; 294‐67001). For immunohistochemistry, antigen retrieval was first performed with Tris/EDTA (pH 8.4), and the sections were incubated with primary antibodies along with 3% H_2_O_2_ to eliminate endogenous peroxidase activity, followed by incubation with peroxidase‐conjugated secondary antibody (Histofine Simple Stain MAX PO; Nichirei Biosciences, Tokyo, Japan). Positive reactions were detected with 3,3′‐diaminobenzidine (Nichirei Biosciences). Sections were counterstained with hematoxylin.

To detect the bone matrix distribution, angiogenesis, and osteoclasts and osteoblasts in the new bone, we analyzed the expression of cell‐specific biomarkers in these tissues. OPN, OCN, RUNX2, and TRAP positivity were evaluated based on the number of cells exhibiting strong immunolabeling of the cell membrane and/or cytoplasm according to the previous studies (Hawthorne et al., 2013; Karsenty, Kronenberg, & Settembre, 2009; Kern, Shen, Starbuck, & Karsenty, 2001; Komori et al., 1997; Oryan et al., 2019; Pereira et al., 2017; Wang et al., 2019). VEGF expression was assessed based on the number of cells bordering new vessels as previously described (Soini et al., 2001). We did not analyze areas into which VEGF‐positive cells were recruited as a result of secondary effects such as tissue necrosis or infection. Anti‐collagen I immunoreactivity and ALP expression were classified as negative (−), weakly to moderately positive (+), or strongly positive (++). Immunolabeled cells were counted under a microscope at 200× magnification by pathologists blinded to any information associated with the samples. Average numbers were calculated from the counts of three areas in each sample. For the comparison, we assessed the immunolabeling patterns of several commercially available antibodies in normal bone of rats that did not undergo surgery and disk implantation.

### 2.8 Statistical analysis

Values are reported as mean ± standard error. Differences between groups were evaluated by one‐way analysis of variance (ANOVA) or the Kruskal–Wallis test. If the ANOVA yielded a significant result, the Tukey–Kramer multiple comparisons test was performed. *p* < .05 was considered to indicate statistically significant differences.

## 3 RESULTS

### 3.1 Radiography and *μ*‐CT examination

**Figure 3:**
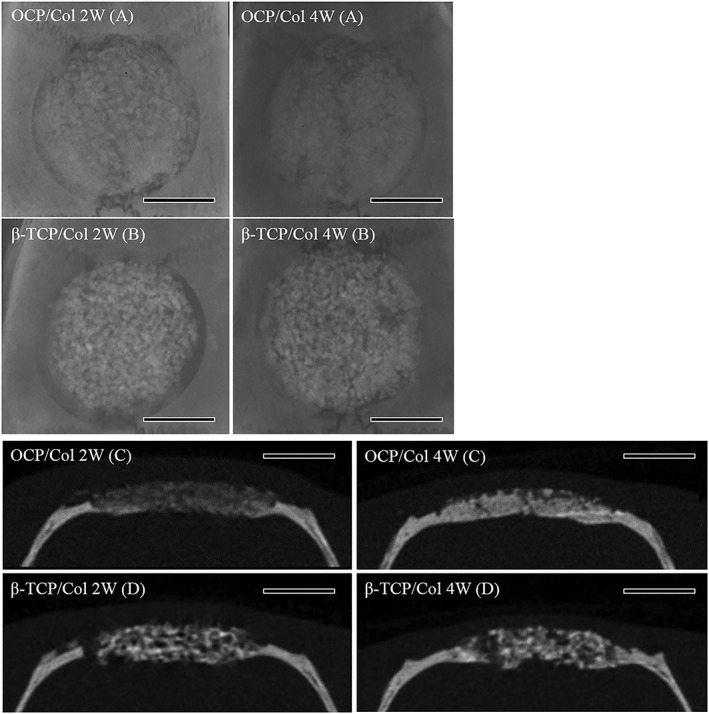
Radiographic examination and micro‐computed tomography (*μ*‐CT) of implants. a, For octacalcium phosphate collagen composite (OCP/Col) radiographic image, thorn‐like radiopaque masses were scattered throughout the defect at week 2, whereas by week 4, the defects were smooth and relatively uniform. b, In *β*‐tricalcium phosphate collagen composite (β‐TCP/Col), isolated granulous radiopacity was observed in the defect after 2–4 weeks. c, In μ‐CT of OCP/Col, slight granulous radiopacities were scattered throughout the defect 2 weeks after implantation. At 4 weeks, the radiopacity in the defect was markedly increased. d, In the β‐TCP/Col group CT image, the isolated granulous radiopacity was intermingled with nonradiopaque areas at weeks 2 and 4, and calcified tissue with uneven shape was observed throughout the experimental period. Scale bar: 4 mm

The results of the radiographic and the *μ*‐CT examination showed that in the OCP/Col group, thorn‐like radiopaque masses were scattered throughout the defect in week 2, with a relatively uniform increase in opacity at week 4 (Figure 3). In the *μ*‐CT image at 2 weeks, the radiopaque figure—which reflected the density of the original bone—extended from the defect margin (mainly from the side of the dura mater) towards the central area of the defect. At 4 weeks, radiopacity was further enhanced in the defect, and its density was similar to that of the original bone, although only slight granulous radiopacities were scattered throughout the defect. In the *β*‐TCP/Col group, the isolated granulous radiopacity was intermingled with nonradiopaque areas at weeks 2 and 4. The granulous radiopacity in the *β*‐TCP/Col group remained irregular shaped and almost immobile throughout the experimental period.

### 3.2 Histological analysis

**Figure 4:**
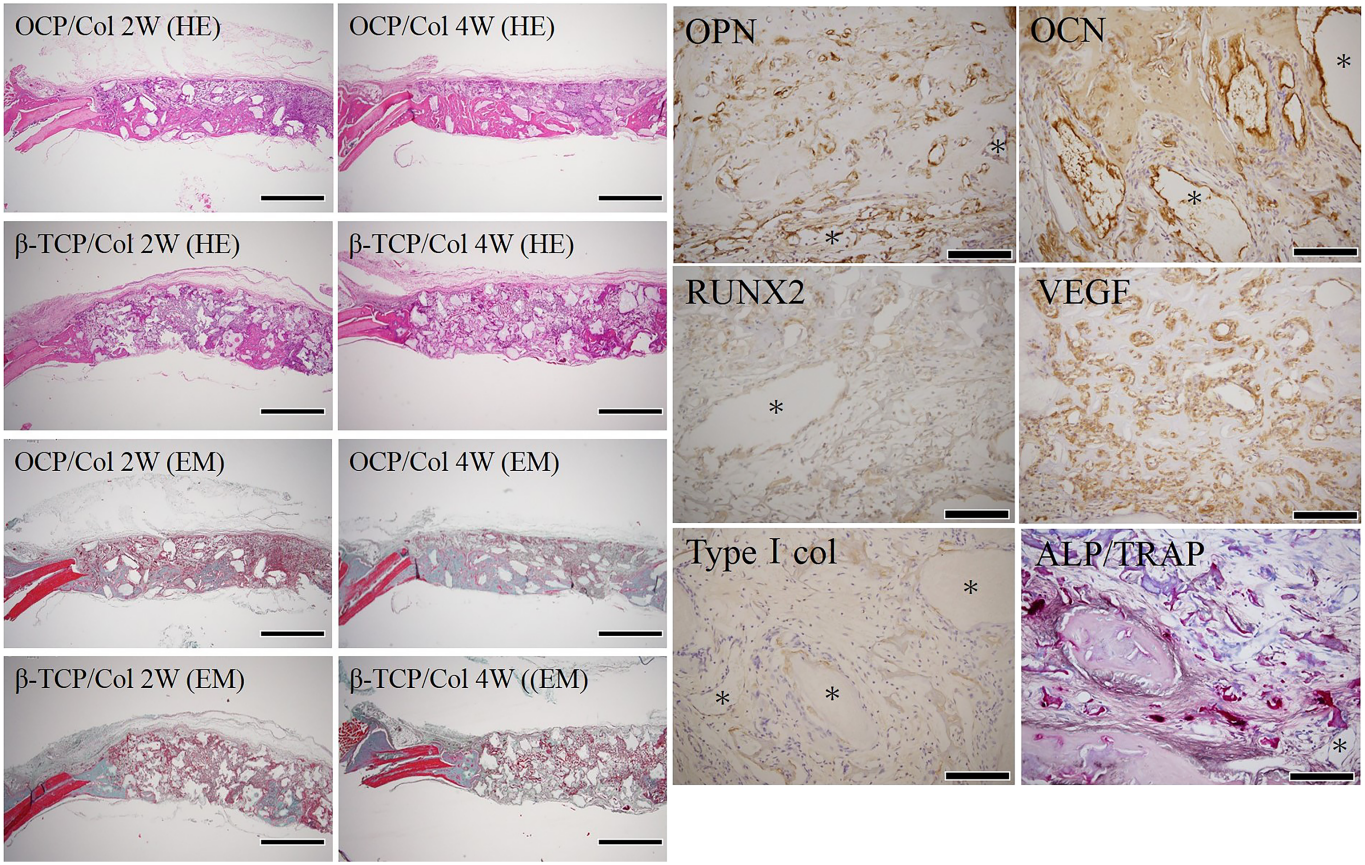
Histological and immunohistochemical analysis of regenerated bone in critical‐size bone defects. In the hematoxylin and eosin (HE) examination, in the octacalcium phosphate collagen composite (OCP/Col) group, relatively uniform eosinophilic new bone was confirmed from the center of the dural surface to the edge of the bone defect. Granule tissue was present around the new bone, and there was no sign of infection. At 2 weeks after surgery, in the OCP/Col group there was elastica‐Masson (EM) staining, collagen fibers were abundant, and bone matrix maturation was observed 4 weeks after surgery. In the control group, an irregular cavity where *β*‐tricalcium phosphate collagen composite (*β*‐TCP/Col) granules had been present persisted without becoming smaller even 4 weeks after surgery, and slight new bone formation was observed around it. Bone formation was slow and focal; collagen fiber formation was also slow. Magnification, 12.5×. Scale bar: 2 mm.Osteopontin (OPN), osteocalcin (OCN), Runt‐related transcription factor 2 (RUNX2), vascular endothelial growth factor (VEGF), and collagen I expression and alkaline phosphatase/tartrate‐resistant acid phosphatase (ALP/TRAP) staining of critical bone defects. Expression of OPN, a marker of osteoblasts and osteoclasts, was also observed on the surface of dura calvaria and on connective tissue of OCP granules and periosteal bone. Expression of the osteoblast‐specific biomarker OCN was detected in the newly formed calvarial bone and the surrounding connective tissue extracellular matrix. RUNX2, a transcription factor important for osteoblast differentiation, was observed in the newly formed calvarial bone cells and the extracellular matrix. Type I collagen, a major bone matrix protein, was expressed throughout the new bone matrix. VEGF was diffusely expressed in the connective tissue around the bone matrix indicating angiogenesis. Asterisks (*) indicate OCP granules. Magnification, 200×. Scale bar: 200 μm [Colour figure can be viewed at http://wileyonlinelibrary.com]

HE and EM staining showed new bone formation at the calvarial dura mater surface and around acidophilic granules in the OCP/Col group at 2 weeks postimplantation along with an abundance of collagenous fibers containing cells; at 4 weeks, bone matrix increased within collagenous fibers (Figure 4). New bone formation was also observed in the β‐TCP/Col group, albeit to a lesser degree, and less bone matrix; and fewer collagenous fibers were formed than in the OCP/Col group.

### 3.3 Immunohistochemistry and ALP/TRAP staining

OPN, OCN, RUNX2, VEGF, collagen I, and ALP/TRAP were expressed in the newly formed bone of OCP/Col samples at 2 weeks postsurgery (Figure 4). OPN‐positive cells were present at the calvarial surface and in OCP granules and the connective tissues around the new bone. OCN immunoreactivity was observed in cells embedded in the new calvarial bone and the surrounding tissues or extracellular matrix. RUNX2, a transcription factor essential for osteoblast differentiation and involved in the production of collagen I in bone tissue, was expressed on the plasma membrane of the newly generated calvarial bone cells and in the extracellular matrix surrounding new bone. The endothelial cell marker VEGF was diffusely expressed in the new bone matrix and soft tissue, suggesting the emergence of vessels. Collagen I, the main bone matrix protein, was ubiquitously expressed throughout the cells and in the extracellular matrix. Positive staining for ALP, a marker of osteoblasts, was detected in the membrane of cells within the new bone, suggesting the presence of osteoblasts. In the OCP/Col group, many TRAP‐positive cells were present in the new calvarial bone, suggesting a substantial osteoclast population.

At 2 weeks after surgery, a quantitative analysis of OPN, OCN, RUNX2, VEGF, and collagen I immunoreactivity and ALP/TRAP staining was performed in the area from the healthy bone margin or the calvarial dura mater surface (Table 1). There was no difference in the expression of any of the above markers at the defect site; however, OPN, OCN, RUNX2, and TRAP—which are related to the development and differentiation of osteoclasts and osteoblasts—tended to be upregulated close to the healthy bone margin or the calvarial dura mater surface.

**Table 1:** Immunohistochemical and alkaline phosphatase/tartrate‐resistant acid phosphatase (ALP/TRAP) staining analysis by dividing the defect area into small compartments

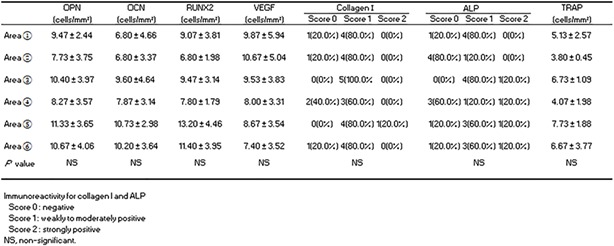

Abbreviations: OPN, osteopontin; OCN, osteocalcin; RUNX2, Runt‐related transcription factor 2; VEGF, vascular endothelial growth factor.

OPN, OCN, RUNX2, VEGF, and collagen I immunoreactivity and ALP/TRAP staining did not differ significantly in the OCP/Col group at 2 and 4 weeks after surgery, although expression tended to be higher at the earlier time point (Table 2). Moreover, all markers were expressed at higher levels in the OCP/Col group than in the *β*‐TCP/Col group at 2 and 4 weeks (*p* < .05 or.01).

**Table 2:** Immunohistochemical and alkaline phosphatase/tartrate‐resistant acid phosphatase (ALP/TRAP) staining analysis compared with *β*‐tricalcium phosphate collagen composite (*β*‐TCP/Col) group compartments

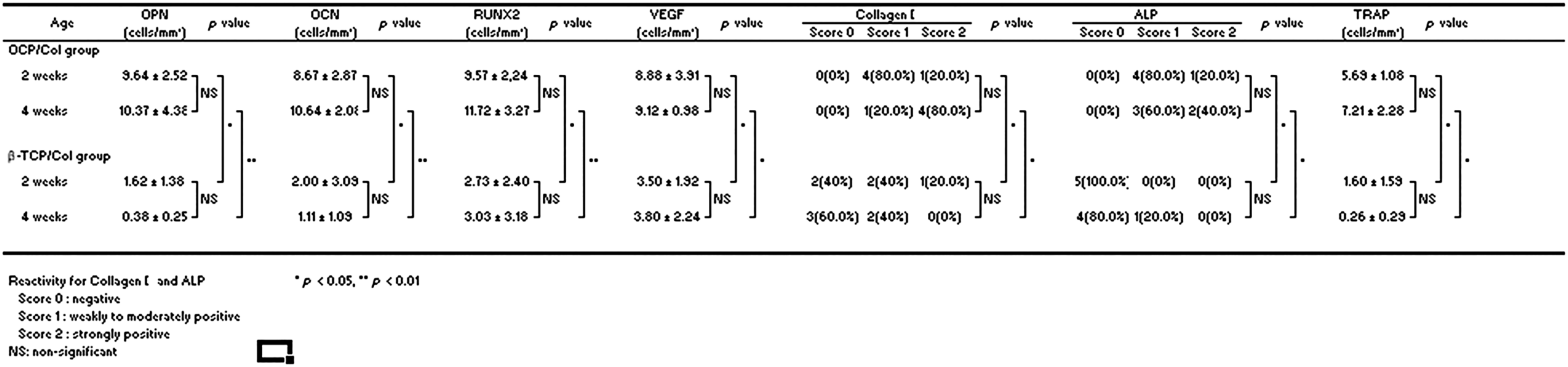

Abbreviations: OCP/Col, octacalcium phosphate collagen composite; OPN, osteopontin; OCN, osteocalcin; RUNX2, Runt‐related transcription factor 2; VEGF, vascular endothelial growth factor.

## 4 DISCUSSION

In this study, we demonstrated, by immunohistochemistry and enzyme histochemistry, that multiple biomarkers related to bone tissue generation were expressed in the newly regenerated bone replacing the OCP/Col disk.

During bone repair, osteoprogenitor cells invade the lesion area along with the new blood vessels. Blood vessel growth not only establishes local circulation in regenerated bone, thereby providing nutrients and oxygen, but also directly promotes bone formation (Kusumbe, Ramasamy, & Adams, 2014; Maes et al., 2010). We found that regardless of the distance from the remaining bone, vascular hyperplasia—as evidenced by VEGF expression—was present in the early phase of bone healing, indicating that the blood vessel growth related to OCP/Col is induced throughout the bone defect, which is an important first step in bone formation that allows the new blood vessels and the osteogenic cells to enter the graft from the transplantation matrix.

OCN, OPN, or RUNX2 are expressed during osteoblast differentiation (Suzuki et al., 2014). OPN is a small integrin binding N‐linked glycoprotein (SIBLING) protein that is a component of the extracellular matrix of bone and dentin and plays a key role in the mineralization of these tissues (Ducy, Zhang, Geoffroy, Ridall, & Karsenty, 1997). OPN biosynthesis occurs in a variety of cell types including preosteoblasts, osteoblasts, osteocytes, odontoblasts, some bone marrow cells, hypertrophic chondrocytes, dendritic cells, and macrophages (Wang & Denhardt, 2008). OCN is a noncollagenous protein hormone found in bone matrix and dentin that is secreted by osteoblasts and is thought to play a role in bone mineralization and calcium ion homeostasis; thus, OCN is proosteoblastic (Mizokami, Kawakubo‐Yasukochi, & Hirata, 2017) and is often used as a marker for bone formation. RUNX2 is essential for osteoblast differentiation and skeletal morphogenesis; it acts as a scaffold for nucleic acids and regulatory factors involved in skeletal gene expression and is the first transcription factor that determines the commitment to an osteoblast fate (Komori, 2017). Runx2 was also suggested to play a key regulatory role in cell cycle entry and exit in osteoblasts (Lucero et al., 2013). ALP is an enzyme present in the membrane of osteoblasts and chondrocytes. In the present study, osteoblast biomarkers were expressed in the new calvarial bone of in rats implanted with OCP/Col; in particular, RUNX2—which is expressed from the early stage of osteoblast differentiation—was strongly expressed at 2 week postsurgery. This suggests that the osteoblast differentiation was normally induced by OCP/Col, leading to active bone matrix formation.

OPN binds to osteoclasts via integrin αvβ3 and contributes to the demineralization and the decomposition of organic components by adhering to the bone (Faccio et al., 1998; Horton, Nesbit, & Helfrich, 1995). Osteoclasts were observed in our study by immunohistochemical detection of OPN and enzyme histochemical analysis of the TRAP activity. A previous study in which OCP was implanted in the granule form in a mouse calvarial critical‐sized defect showed that the bone formation increased with granule size, which was accompanied by a greater number of TRAP‐positive cells (Murakami, Honda, Anada, Shimauchi, & Suzuki, 2010). We detected many OPN‐ and TRAP‐positive osteoclasts at the center of the bone defect away from the residual healthy bone throughout the experiment. Thus, OCP/Col induces rapid bone remodeling without being influenced by the residual healthy bone.

Type I collagen is the most abundant collagen in the human body; it is present in scar tissue and is the end product of tissue repair. In bone, type I collagen accounts for 90% of the total bone matrix protein (Ricard‐Blum & Ruggiero, 2005). We found that type I collagen was expressed throughout the bone defect, with high levels observed 4 weeks after surgery. These results suggest that OCP induces the secretion of a bone matrix composed of type I collagen by osteoblasts, leading to the formation of uncalcified osteoid.

An interesting finding in the present study is that the expression of all markers of bone formation was higher in rats implanted with OCP/Col than in those implanted with *β*‐TCP/Col, indicating that bone formation, which includes angiogenesis, differentiation of osteoclasts and osteoblasts, and bone matrix consolidation, was more strongly promoted in the former group.

Osteoinduction, osteoconduction, and osteogenesis are important properties of bone grafting materials. Osteoconductive materials serve as a scaffold for new bone growth perpetuated by native bone; osteoblasts from the margin of the defect being grafted utilize this material as a framework upon which to spread and generate the new bone. Osteoconduction involves the recruitment of immature cells and their development into preosteoblasts. Osteogenesis occurs when osteoblasts originating from the bone graft material contribute to new bone growth along with bone generated via the other two mechanisms (Frame, 1987; Lane, 1995; Misch & Dietsh, 1993). Although autologous bone grafting exhibits these three features, existing synthetic bioresorbable materials generally only possess osteoconductive capacity. A bone graft material that is both osteoconductive and osteoinductive can not only function as a scaffold for existing osteoblasts but can also trigger the differentiation of the new osteoblasts, thereby accelerating graft integration.

A previous radiographic analysis showed that the newly formed bone is regenerated throughout the defect upon OCP/Col implantation (Kamakura et al., 2004; Kamakura et al., 2002; Kamakura et al., 1997). In our study, μ‐CT, radiography, and HE and EM staining revealed that the bone regeneration induced by OCP/Col is not restricted to the defect margin but occurs almost uniformly throughout the defect. An interesting finding from the immunohistochemistry and enzyme histochemistry experiments is that bone regeneration induced by OCP/Col—including the development and the differentiation of osteoclasts or osteoblasts, vessel proliferation, and bone matrix formation—proceeds irrespective of the proximity to healthy bone margin or dura calvaria. This suggests that the induction of bone regeneration by OCP/Col does not depend on the residual healthy tissue; that is, OCP/Col is not only osteoconductive (allowing newborn blood vessels and osteogenic cells from the defect site tissue to invade into the bone defect), but also osteoinductive (producing undifferentiated mesenchymal cells and inducing the differentiation of osteogenic cells and the formation of the bone matrix).

## 5 CONCLUSIONS

OCP/Col implantation in a critical‐sized calvarial defect in rat stimulated bone regeneration (as evidenced by the accumulation of osteoblasts and osteoclasts, regardless of the distance from the remaining bone), including at the center of the defect. Our results indicate that OCP/Col is a bone regenerative material with osteoconductive capacity that depends on the residual healthy bone tissue as well as the osteoinductive capacity, which allows angiogenesis and invasion of osteogenic cells from host tissue into the bone defect. These findings indicate that OCP combined with collagen can effectively promote the repair of bone defects via two distinct processes.

## CONFLICT OF INTEREST

One of the authors (S.K.) has obtained a patent for OCP/Col in Japan (#5046511). The other authors have no conflicts of interest to declare in relation to this work.
